# Tuna Oil‐Enriched Toddler Formula Enhances DHA Status in Indonesian Toddlers

**DOI:** 10.1002/fsn3.70838

**Published:** 2025-09-05

**Authors:** Diana Sunardi, Samaneh G. Fard, Mia Puspita Ratih, Aprilia Herawati, Glenn Elliott, Andrew J. Sinclair

**Affiliations:** ^1^ Department of Nutrition Faculty of Medicine Universitas Indonesia‐Cipto Mangunkusumo Hospital Jakarta Pusat Indonesia; ^2^ Nu‐Mega Ingredients Pty Ltd. Brisbane Australia; ^3^ Indonesian Nutrition Association Jakarta Pusat Indonesia; ^4^ Faculty of Health Deakin University Burwood Australia; ^5^ Department of Nutrition Dietetics and Food, Monash University Notting Hill Australia

**Keywords:** bioavailability, docosahexaenoic acid, fortified formulas, microencapsulation, omega‐3 fatty acids, toddler nutrition

## Abstract

Docosahexaenoic acid (DHA) is essential for brain and cognitive development in toddlers; however, global intakes often fall below recommended levels. This study evaluated the bioavailability of DHA from commercial toddler formulas fortified with either microencapsulated high‐DHA fish oil powder or high‐DHA fish oil. A double‐blind, randomized controlled trial was conducted in 120 healthy Indonesian toddlers aged 2–3 years. Participants were assigned to one of three groups: (A) dry‐blended formula with microencapsulated high‐DHA fish oil powder, (B) unfortified control formula, or (C) wet‐mixed high‐DHA fish oil formula. Bioavailability was assessed using both blood and fecal fatty acid levels. Both DHA‐fortified formulas increased blood DHA levels, total omega‐3 fatty acids, and the Omega‐3 Index. However, only the microencapsulated DHA formula led to statistically significant improvements compared with the control. Apparent DHA digestibility and the incremental area under the curve (iAUC) for blood DHA were significantly higher in the microencapsulated DHA formula group compared with the high‐DHA fish oil formula group. Adherence and intake were also highest in the microencapsulated group, possibly due to improved palatability, although sensory characteristics were not directly assessed. While improvements in DHA status were statistically significant, the overall magnitude of change was modest, and its clinical significance remains uncertain. Nonetheless, microencapsulation may offer advantages for enhancing DHA bioavailability in young children. Higher DHA dosages or longer intake durations may be needed to achieve Omega‐3 Index levels exceeding 8% in populations with low baseline status.

## Graphical abstract

This double‐blinded randomized trial assessed the effect of different DHA fortification methods in toddler formula on blood DHA levels, digestibility, and adherence. Among 120 toddlers, microencapsulated high‐DHA tuna oil powder added via dry‐blending (Group A) significantly improved blood DHA levels compared to the unfortified control. DHA digestibility remained high in both fortified groups, with Group A also achieving the best adherence.

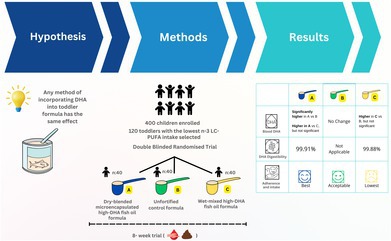

**Abbreviations**
ALA: alpha‐linolenic acid
ANOVA: analysis of variance
ARA: arachidonic acid
DHA: docosahexaenoic acid
DPA: docosapentaenoic acid
EFSA: European Food Safety Authority
EPA: eicosapentaenoic acid
FFQ: Food Frequency Questionnaire
GOED: Global Organization for EPA and DHA
iAUC: incremental area under the curve
LNA: linoleic acid
n‐3 LC‐PUFA: omega‐3 long‐chain polyunsaturated fatty acids
n‐6:n‐3: omega‐6/omega‐3
n‐6 PUFA: omega‐6 polyunsaturated fatty acids
SE: standard error
W: week (e.g., W0 = Week 0)

## 1 Introduction

Infants and young children undergo rapid growth and development, particularly during the transition from complementary feeding at 12 months to a mixed diet at 36 months (Ghasemi Fard et al. 2019). Omega‐3 long‐chain polyunsaturated fatty acids (n‐3 LC‐PUFA), particularly docosahexaenoic acid (DHA), are essential for brain, retinal, and cognitive development during early childhood (Ghasemi Fard et al. 2019). However, access to DHA‐rich foods such as fatty fish is often limited due to dietary, environmental, or cultural factors, resulting in suboptimal DHA intake globally.

DHA intake among toddlers remains below recommended levels in most countries (Meyer et al. 2003; Gibson and Sidnell 2014; Keim and Branum 2015; Innis et al. 2004; Barbarich et al. 2006; Kim et al. 2019; Tsuboyama‐Kasaoka et al. 2013). The European Food Safety Authority (EFSA) recommends daily DHA intakes of 100 mg for children under 24 months and 250 mg for older children (2–18 years) (EFSA Panel on Dietetic Products, Nutrition, and Allergies 2014). DHA fortification of infant and toddler formulas has been widely adopted to address this gap.

A previous study demonstrated that microencapsulation of high‐DHA tuna oil powder improved DHA bioavailability in Malaysian toddlers, although it used experimental rather than commercial formulas (Ghasemi Fard et al. 2020). The present study directly compared two DHA incorporation strategies in commercially available formulas: dry‐blended microencapsulated high‐DHA fish oil powder (added after the spray‐drying process) versus wet‐mixed high‐DHA fish oil. In the wet‐mixing method, ingredients are combined in a liquid phase, and fish oil is added before or after heat treatment prior to spray‐drying. To date, no previous studies have compared the impact of these manufacturing methods on DHA bioavailability in toddlers, making this a novel investigation.

In nutrition science, “bioavailability” lacks a standardized definition and often overlaps with “digestibility” (Ghasemifard et al. 2014). For n‐3 LC‐PUFA, bioavailability is typically assessed through blood levels after omega‐3 consumption, while excretion is rarely considered. Blood n‐3 LC‐PUFA levels are influenced by complex processes such as digestion, absorption, metabolism, and β‐oxidation, leading to variable fatty acid deposition (Ghasemifard, Hermon, et al. 2015; Ghasemi Fard et al. 2014, 2021). External factors, including dietary fat content (Ghasemifard et al. 2014), oil source (Ghasemifard, Hermon, et al. 2015), and dosing regimen (Ghasemifard, Hermon, et al. 2015; Ghasemifard, Sinclair, et al. 2015), also affect bioavailability. Given these variables, blood levels alone may not reliably indicate true bioavailability. Therefore, in this study, we assessed both blood fatty acid concentrations and fatty acid excretion.

Fortifying foods with n‐3 LC‐PUFA presents challenges, as these fatty acids are susceptible to oxidation, producing free radicals and unpleasant odors. Microencapsulation has emerged as a preferred strategy to protect n‐3 LC‐PUFA (Barrow et al. 2007; Kampa et al. 2007) and has been widely incorporated into infant and toddler formulas (Bozin et al. 2007; Wang et al. 2018). Despite these advances, some manufacturers still use direct fish oil addition due to cost considerations and/or industry practices.

This study aimed to compare the bioavailability of DHA between dry‐blended microencapsulated high‐DHA tuna oil powder formula and wet‐mixed high‐DHA tuna oil formula in healthy Indonesian toddlers aged 2–3 years over 8 weeks. This research extended previous findings from Malaysian toddlers (Ghasemi Fard et al. 2020) and aimed to enhance understanding of cross‐population differences in fatty acid profiles and DHA bioavailability.

## 2 Materials and Methods

### 2.1 Study Design and Ethical Approval

This study was a two‐phase, double‐blind, randomized controlled trial conducted among toddlers in East Jakarta, Indonesia. Ethics approval was obtained from the Ethics Committee of the Faculty of Medicine, Universitas Indonesia (KET‐301/UN2.F1/ETIK/PPM.00.02/2020). The study received initial approval on 16 March 2022, with subsequent modifications approved on 1 March 2023 and 17 April 2024. The study was registered at ClinicalTrials.gov (NCT04460287; registered on 7 January 2020). Written informed consent was obtained from parents or guardians of all participants.

### 2.2 Participants and Screening

Toddlers aged 2–3 years were recruited from local integrated health centers in East Jakarta, Indonesia. In Phase I (screening for habitual omega‐3 intake), mothers of 400 healthy toddlers were initially invited to complete a structured Food Frequency Questionnaire (FFQ) designed to assess habitual dietary intake of n‐3 LC‐PUFA. Mothers completed the questionnaire in the presence of a trained research assistant, who provided clarification if needed and ensured all questions were answered. The FFQ was a semiquantitative tool adapted for toddlers under five, intended to capture a wide range of local dietary patterns and food preparation methods. It was developed based on previously validated work by Hartriyanti et al. (2023) and informed by FAO dietary assessment guidelines for low‐resource settings (FAO 2018).

**FIGURE 1:**
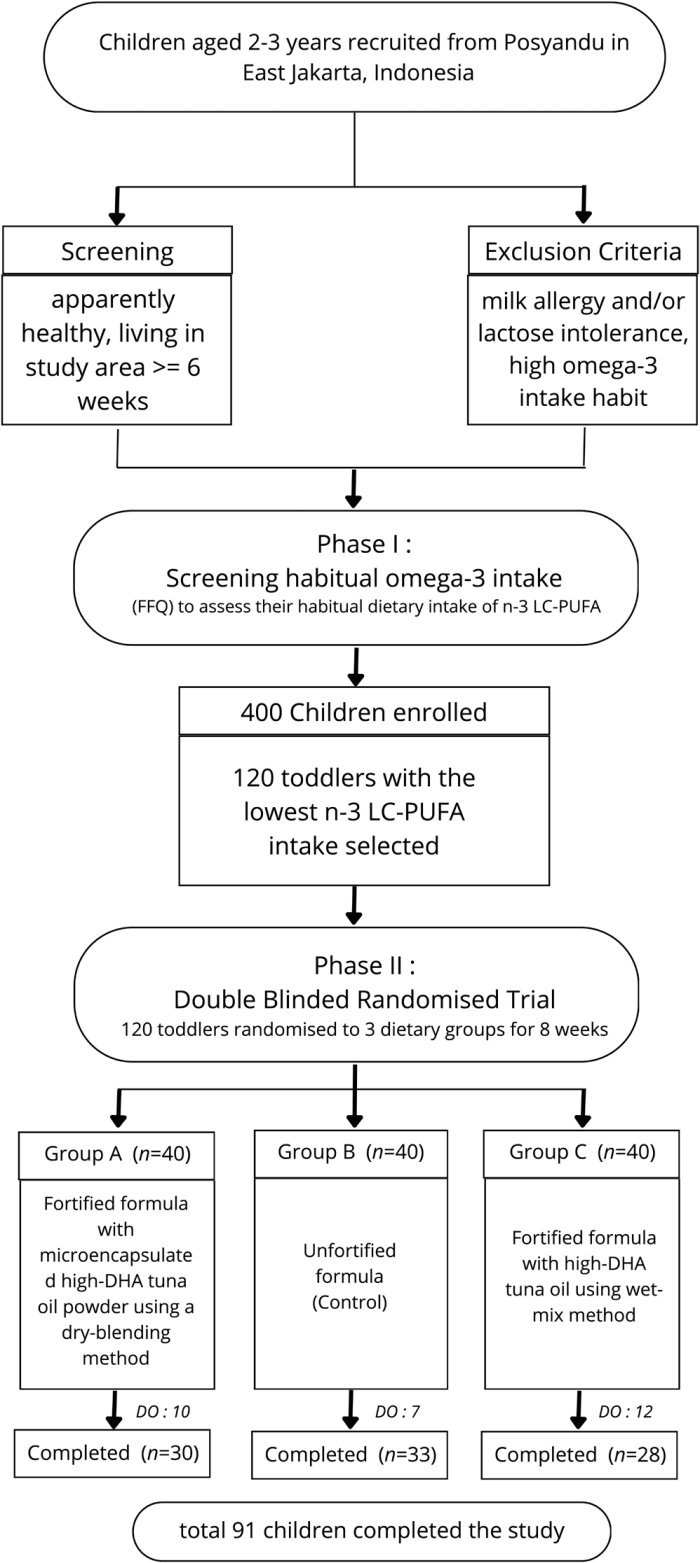
Study flow diagram of participant screening, randomization, and completion. Group A: Dry‐blended formula with microencapsulated high‐DHA fish oil powder; Group B: Unfortified control formula; Group C: Wet‐mixed formula with high‐DHA fish oil.

Inclusion criteria included healthy boys and girls residing in the study area for at least 6 weeks, with no milk allergies, lactose intolerance, or habitual high omega‐3 intake. Based on the FFQ results, 120 toddlers met the eligibility criteria and were selected to proceed to Phase II of the study (Figure 1).

Parents were instructed to avoid providing omega‐3 rich foods to their children for 2 weeks before the intervention. Restricted foods included marine‐origin products (fresh fish, canned fish, fish paste, and shellfish), sea vegetables (seaweed), walnuts, avocados, egg yolks, omega‐3 fortified products, chia seeds, cod liver oil, and fish oil supplements. Dropout was defined as missing more than three consecutive days of formula intake.

### 2.3 Randomization and Blinding

In Phase II, eligible toddlers were randomly assigned to one of three groups (*n* = 40 per group) using a research‐grade randomization tool. The randomization sequence was generated by an independent researcher who was not involved in participant recruitment, group assignment, or outcome assessment, in order to ensure allocation concealment. To maintain blinding and minimize bias, a strict labeling and distribution protocol was followed. Two research assistants, who were not involved in outcome analysis, labeled the formulas as group A, B, or C, placed them into identical containers, and delivered them to the research center. All individuals involved in the study, including participants, their families, data collectors, chemical analysis technicians, and research staff were blinded to group allocation throughout the trial.

### 2.4 Intervention

The intervention formulas were commercially available toddler products purchased through retail channels in Indonesia. Based on proprietary knowledge from Nu‐Mega Ingredients Pty Ltd., the formulas used for groups A and C were known to be fortified with either microencapsulated high‐DHA tuna oil powder (group A) or high‐DHA tuna oil (group C), both supplied by Nu‐Mega Ingredients Pty Ltd. Brand names of the products are not disclosed to maintain commercial confidentiality.

The intervention formulas and their nutritional contents varied across the groups as reported in Table 1. The initial aim was to provide toddlers with a higher intake of DHA and EPA, in line with EFSA recommendations. However, due to limitations of available commercial formulas with this fortification rate, the target DHA intake was adjusted to 50 mg per day to ensure practicality and compliance.

**TABLE 1:** Nutrition composition of intervention formulas.

	Group A	Group B	Group C
Serving size (g)	40	40	31.65
Servings per day	2	2	3
Nutrition compositions per serving^a^			
Energy (Kcal)	170	160	150
Protein (g)	6	5	5
Carbohydrate (g)	25	16	19
Fat—total (g)	6	3	6
Saturated (g)	NS	1	NS
Monounsaturated fats (g)	NS	NS	NS
Polyunsaturated fats (g)	NS	NS	NS
LNA (mg)	800	320	1076
ALA (mg)	124	37	129
ARA (mg)	NS	NS	16
EPA (mg)	NS	NS	NS
DHA (mg)	25	0	16
DHA (mg/day)	50	0	48

*Note:* Group A: dry‐blended formula with microencapsulated high‐DHA fish oil powder; Group B: unfortified control formula; Group C: wet‐mixed formula with high‐DHA fish oil.

Abbreviations: ALA, alpha‐linolenic acid; ARA, arachidonic acid; DHA, docosahexaenoic acid; EPA, eicosapentaenoic acid; LNA, linoleic acid; NS, not specified on the formula labels.

^a^ As reported on formula labels.

Parents collected the labeled, unopened formula from the research center. After 1 week of consumption, any remaining formula was returned in its original containers, weighed to assess leftover amounts, and exchanged for a new supply with the same code. Parents were asked to freshly prepare the milk drink daily by measuring 200 mL of warm water into a bottle, adding one level scoop (40 g for groups A and B, and 31.65 g for group C) of formula, and shaking the mixture.

### 2.5 Sample Collection

Blood samples were collected from toddlers' thumbs using an automatic lancing device at baseline (W0), and at weeks 2, 4, 6, and 8. The device was included in the dried blood spot kit provided by OmegaQuant Analytics (Sioux Falls, USA). Anthropometric data were collected during screening, at baseline, and at week 8.

To support study compliance and logistics, one community health assistant was assigned to approximately every 15 participants, resulting in a total of eight assistants supporting the enrolled toddlers. The study also involved four nutrition data collectors, two chemical analysis technicians, and two formula labeling assistants.

### 2.6 Blood Fatty Acid Measurement

The OmegaQuant dried blood spot (DBS) method was selected for its noninvasive and cost‐effective suitability in children. Capillary blood was collected via finger prick and applied to the designated collection area of the DBS card, which had been pretreated with an antioxidant cocktail (FAPS) to stabilize polyunsaturated fatty acids (PUFAs), including EPA and DHA. Samples were labeled with the participant's identification number, air‐dried at room temperature for 2 h, and stored at −20°C.

According to previously published validation data (Harris and Polreis 2016), EPA and DHA in these antioxidant‐treated DBS samples are stable for at least 6 weeks at room temperature, 4 weeks refrigerated, 3 years at −20°C, and 4 years at −80°C. After collection, DBS cards were transferred to a storage facility at the Indonesian Nutrition Association in Jakarta, Indonesia, and refrigerated at 4°C to preserve sample integrity under conditions of high environmental temperature and humidity. All samples were subsequently shipped to OmegaQuant (Sioux Falls, USA) at designated time points for fatty acid analysis.

Fecal samples were collected daily, placed in zip‐lock plastic bags, and stored at −20°C. For each participant, the fecal samples collected over 1 week were weighed, pooled, and homogenized. Two 1‐g aliquots of the homogenized sample were sent to OmegaQuant (Sioux Falls, USA) for analysis. Baseline feces (W0) were collected 1 day prior to the intervention (day −1). Fecal data were averaged over 2‐week intervals and reported at the end of each interval (i.e., weeks 1–2 as week 2, weeks 3–4 as week 4, weeks 5–6 as week 6, and weeks 7–8 as week 8).

### 2.7 Data Analysis

Based on the previous study (Ghasemi Fard et al. 2020), a sample size of 15 per group was sufficient to detect a significant difference in the main outcome with at least 80% power. For this study, we targeted 25–30 participants per group to achieve 95% power. To accommodate an anticipated dropout rate of up to 20%, due to the intensive nature of the study (e.g., fecal collection) and its implementation in low‐income communities, the final sample size was increased to 40 participants per group.

Results were expressed as mean ± standard error (SE). Differences between treatment groups over time were analyzed using a two‐way analysis of variance (ANOVA), evaluating the effects of treatment, time, and their interaction. Where significant effects were observed, post hoc comparisons were conducted using Tukey's test, with a significance threshold set at *p* < 0.05.

To account for variability in formula intake, biweekly formula consumption and corresponding DHA intake were included as covariates in the ANOVA models, thereby controlling differences in DHA intake across the groups. Additional outcome parameters—relative bioavailability and apparent DHA digestibility—were calculated to provide a comprehensive assessment of DHA utilization.

Relative bioavailability of DHA was calculated by dividing the absolute change in blood DHA (% of total fatty acids) from baseline to week 8 by the total DHA intake (mg) over the intervention period, following the method described by Ghasemifard, Hermon, et al. (2015); Ghasemifard, Sinclair, et al. (2015). Results are expressed as % DHA per mg DHA intake:

$$\text{Relative bioavailability of}\;\mathrm{DHA}=\left(\frac{\text{Blood}\;{\mathrm{DHA}}_{8}-\text{Blood}\;{\mathrm{DHA}}_{0}}{\text{Formula intake}}\right)$$

Apparent DHA digestibility was calculated as the percentage of DHA intake not excreted in feces:

$$\text{Apparent}\;\mathrm{DHA}\;\text{digestibility}\;\left(\%\right)=\left(\frac{\mathrm{DHA}\;\text{intake}-\mathrm{DHA}\;\text{excreted}\;}{\mathrm{DHA}\;\text{intake}}\right)x\;100$$

Due to high baseline variability in blood DHA levels, the incremental area under the curve (iAUC) was calculated using the trapezoidal rule. Only positive increments from baseline were included, ensuring that only increases above the baseline contributed to the total area. This approach enabled a more accurate evaluation of the post‐treatment response while minimizing baseline effects. Tukey's post hoc test was applied for pairwise group comparisons where appropriate.

## 3 Results

### 3.1 Subject Characteristics

Out of 120 toddlers enrolled in Phase II, 91 children completed the study: 30 in group A, 33 in group B, and 28 in group C. A total of 29 toddlers withdrew due to various reasons, including mild discomfort (e.g., diarrhea) and a refusal to consume the formula. No allergic reactions or serious adverse events were reported.

Baseline characteristics were comparable across the groups, with no statistically significant differences observed (Table 2). Among the children who completed the study, 47.3% were male and 52.7% female. The mean age was 33.9 ± 8.1 months (range: 12–48 months), with no significant differences in age among the three groups.

**TABLE 2:** Baseline characteristics of study participants by intervention group.

Title 1	Group A	Group B	Group C	*p*
Subjects per group	30	33	28	
Body weight (kg)	12.22 ± 0.58^a^	12.71 ± 0.58^a^	12.34 ± 0.40	*p* = 0.779
Height (cm)	89.1 ± 1.5	91.2 ± 1.33	90.5 ± 1.30	*p* = 0.601
Body mass index	15.3 ± 0.40	15.18 ± 0.48^a^	15 ± 0.30	*p* = 0.831
Age (month)	32.43 ± 1.7	35.88 ± 1.32	33.14 ± 1.35	*p* = 0.202
Gender (*n*)				
Male	14	18	11	*p* = 0.49
Female	16	15	17	

*Note:* Value expressed as mean ± SE.

^a^ Not normally distributed. Group A: dry‐blended formula with microencapsulated high‐DHA fish oil powder; Group B: unfortified control formula; Group C: wet‐mixed formula with high‐DHA fish oil.

### 3.2 Formula Intake

Milk formulas were prepared and consumed according to the study protocol, with parents following group‐specific serving instructions. Formula intake was monitored weekly by weighing leftover formula to accurately quantify actual consumption. Toddlers in group A consumed all of their allocated formula, with intake slightly exceeding the expected amount (101% of planned intake). In contrast, formula intake was lower in groups B and C, averaging approximately 90% in group B and 70% in group C (Table 3). Parents in group C reported that their children disliked the taste of the formula, often describing it as bitter.

**TABLE 3:** Biweekly formula intake (%) and DHA intake from formula (mg).

Time/Group	Group A	Group B	Group C	*p*
Biweekly formula intakes (%)				
W2	101.2 ± 2.2	83.3 ± 8.6	68.9 ± 1.5	< 0.0001
W4	101.1 ± 2.1	89.9 ± 7.1	69.2 ± 1.2	< 0.0001
W6	101.9 ± 1.9	88.7 ± 6.2	70.2 ± 1.9	< 0.0001
W8	100.9 ± 1.7	88.8 ± 6.4	70.7 ± 1.9	< 0.0001
Biweekly DHA intake from formula (mg)				
W2	708.5 ± 15.8	—	463.7 ± 10.3	< 0.0001
W4	707.8 ± 14.7	—	465.8 ± 8.6	< 0.0001
W6	713.7 ± 13.8	—	472.6 ± 13.1	< 0.0001
W8	706.9 ± 12.5	—	475.3 ± 13.3	< 0.0001

*Note:* Value expressed as mean ± SE. Group A: dry‐blended formula with microencapsulated high‐DHA fish oil powder; Group B: unfortified control formula; Group C: wet‐mixed formula with high‐DHA fish oil.

While the reduced intake in group B was less critical due to the absence of DHA in this formula, the lower intake in group C significantly impacted total DHA consumption (Table 3).

### 3.3 Fatty Acid Levels in Whole Blood

At baseline, the mean whole blood fatty acid composition among 91 Indonesian toddlers was approximately 39.3% saturated fat, 25.9% monounsaturated fat, 31.5% n‐6 PUFA, and 2.4% n‐3 PUFA (Table S1). Linoleic acid (LNA, 18:2n‐6) and arachidonic acid (ARA, 20:4n‐6) were the primary components of n‐6 PUFA, comprising approximately 20.3% and 7.3% of total fatty acids, respectively, while DHA was the main component of n‐3 PUFA, accounting for approximately 1.6% of total fatty acids in whole blood (Table S2).

Over the 8‐week intervention, toddlers in groups A and C exhibited a reduction in total saturated fat, particularly in palmitic acid (C16:0), compared to group B, which showed a slight increase. Changes in monounsaturated fat levels were minimal across all groups. Notably, certain long‐chain saturated and monounsaturated fatty acids (e.g., C22:0, C24:0, and C24:1n9) were significantly lower in groups A and C than in group B by the end of the study. For omega‐6 fatty acids, total n‐6 PUFA levels remained relatively stable in groups A and C but showed a slight decline in group B (Table S2).

### 3.4 Omega‐3 Fatty Acids and Omega‐3 Index

The total omega‐3 fatty acids, individual omega‐3 fatty acid levels, and Omega‐3 Index in toddlers fed various formulas over 8 weeks are reported in Table S2. For all 91 Indonesian toddlers who completed this study, the average percentage of total n‐3 PUFA at baseline was 2.4%. The total n‐3 PUFA levels increased significantly within groups A and C, while group B showed a small, non‐significant decrease. Between the groups, at baseline, total n‐3 PUFA levels were similar across all groups. However, from week 2 onward (weeks 2, 4, 6, and 8), total n‐3 PUFA levels in group A and group C were higher than in the control group (group B), with group A showing significantly higher levels than group B. This significant increase was not observed in group C.

For alpha‐linolenic acid (ALA, 18:3n‐3), levels showed an increasing trend within each group, with the highest levels observed at the end of the study for groups A and B, and at week 6 in group C, followed by a drop at week 8.

Eicosapentaenoic acid (EPA, C20:5n‐3) levels increased nonsignificantly in the middle of the study in group A at weeks 2 and 4, and in group C at week 4, before returning to baseline levels by week 8. In contrast, group B showed a decreasing trend in EPA levels, with significantly lower levels observed at weeks 6 and 8. Between the groups, EPA levels in groups A and C were consistently higher than in group B across all time points, with the difference being significant only at week 4.

Docosapentaenoic acid (DPA, C22:5n‐3) levels in both groups A and C showed a small, nonsignificant increase, peaking at week 4 before returning to baseline levels by the end of the study. In contrast, group B exhibited a small but significantly lower DPA level at weeks 6 and 8. Between the groups, no significant differences were observed at any time points.

**FIGURE 2:**
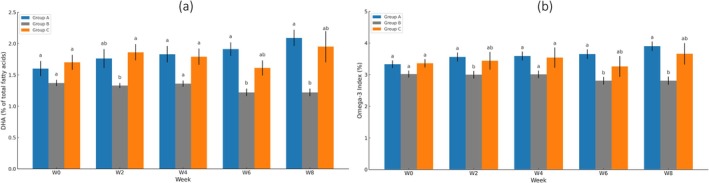
Changes in whole blood DHA levels (a) and Omega‐3 Index (b) over 8 weeks across the three groups. Value expressed as mean ± SE. Groups with different letters are significantly different. Group A: Dry‐blended formula with microencapsulated high‐DHA fish oil powder; Group B: Unfortified control formula; Group C: Wet‐mixed formula with high‐DHA fish oil.

Docosahexaenoic acid (DHA, C22:6n‐3) levels in groups A and C showed a significant increase toward the end of the study. In contrast, group B exhibited a small, nonsignificant decreasing trend in DHA levels. Between the groups, baseline DHA levels were comparable across all three groups. At all time points, DHA levels in groups A and C were higher compared with group B, with a significant difference observed specifically between group A and group B at weeks 6 and 8 (Figure 2a).

The Omega‐3 Index is defined as the sum of EPA and DHA expressed as a percentage of total fatty acids in erythrocytes (Harris 2008). The Omega‐3 Index followed a trend similar to DHA levels. Groups A and C showed a significant increase towards the end of the study, while group B exhibited a nonsignificant decrease (Figure 2b). Between the groups, baseline Omega‐3 Index levels were comparable across all groups. However, Omega‐3 Index levels in groups A and C were consistently higher than those in group B, with a significant difference observed specifically between group A and group B at weeks 2, 6, and 8.

### 3.5 Relative Bioavailability

**FIGURE 3:**
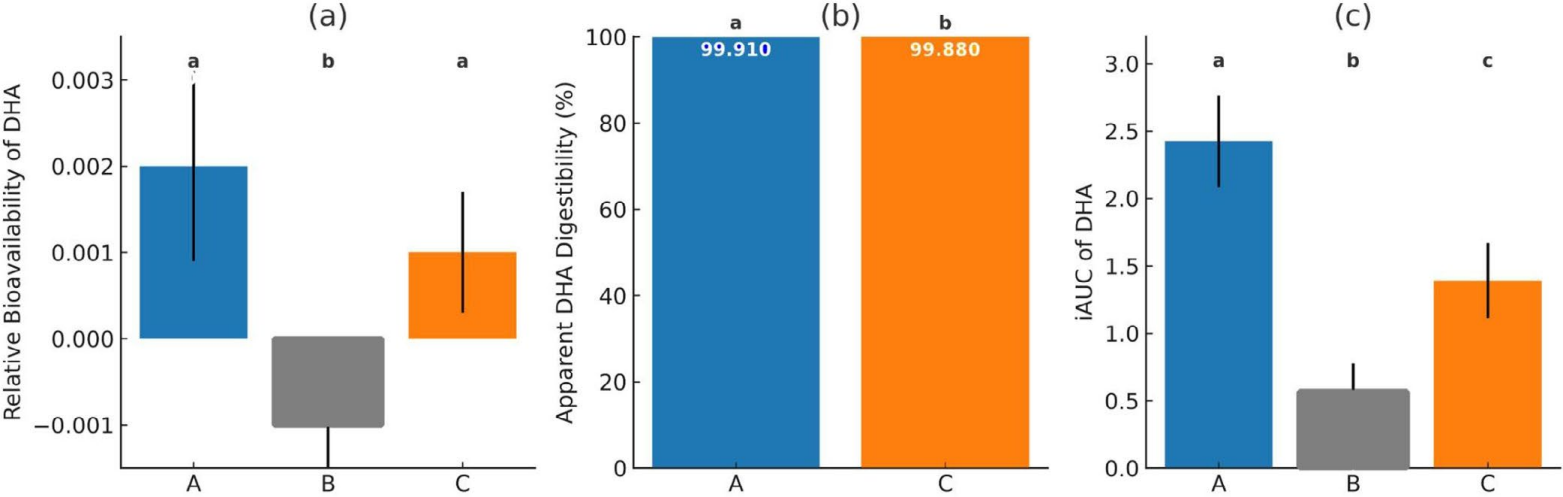
Comparison of relative bioavailability of DHA (a), apparent DHA digestibility (b), and iAUC of blood DHA (c) across the three groups. Value expressed as mean ± SE. Groups with different letters are significantly different. Group A: Dry‐blended formula with microencapsulated high‐DHA fish oil powder; Group B: Unfortified control formula; Group C: Wet‐mixed formula with high‐DHA fish oil.

The relative bioavailability of selected fatty acids (ALA, DPA, DHA, EPA, and ARA) was calculated. The results indicated no significant differences in the relative bioavailability of ARA, ALA, EPA, and DPA among the three groups. However, the relative bioavailability of DHA was significantly higher in groups A and C compared with group B, with no significant differences were observed between groups A and C (Figure 3a).

### 3.6 Apparent DHA Digestibility

Apparent DHA digestibility was calculated, revealing a small but statistically significant difference between the two treatment groups. Group A exhibited an apparent DHA digestibility of 99.91%, while group C showed a digestibility of 99.88%. These findings suggest that both treatment methods achieve near‐complete DHA absorption, with group A demonstrating a marginally higher digestibility rate (Figure 3b).

### 3.7 Incremental AUC (iAUC) for Blood DHA

The iAUC for blood DHA analysis showed significant between‐group differences (*p* < 0.001). Group A had the highest iAUC (2.43 ± 0.36), followed by group C (1.39 ± 0.26), while group B had the lowest (0.57 ± 0.19). Tukey's post hoc test revealed that group A had a significantly higher blood DHA iAUC compared with group B (mean difference = 1.86, *p* < 0.001) and group C (mean difference = 1.04, *p* = 0.029) (Figure 3c).

### 3.8 Fatty Acid Levels in Feces

Daily fecal wet weight and total fecal fat excretion remained consistent across all groups, with no significant differences observed between or within groups at any time point (Table S3). The mean concentrations of selected omega‐6 and omega‐3 fatty acids in fecal samples over the 8‐week period are presented in Table S4. Overall, no substantial differences in fecal omega‐6 fatty acid excretion were observed between groups, with most fatty acids remaining stable or exhibiting nonsignificant fluctuations throughout the study.

Fecal ALA levels decreased nonsignificantly across all groups by week 8, following early increases in groups A and B. EPA levels declined across all groups, with a significant reduction observed only in group B. DPA and DHA levels remained stable, with no significant changes observed within or between groups. Daily excretion of DPA, EPA, and DHA followed the same patterns, with a significant reduction in EPA excretion in groups B and C, and no significant changes for DPA or DHA (Table S4).

## 4 Discussion

This study demonstrated that microencapsulated DHA in toddler formula was bioavailable and led to a modest but significant improvement in blood omega‐3 status in Indonesian toddlers. While both DHA‐fortified formulas increased blood DHA levels, only the microencapsulated DHA formula showed a statistically significant increase compared with the control. A similar pattern was observed for the Omega‐3 Index; although both DHA groups improved over the 8‐week period, only the microencapsulated group differed significantly from the control. Neither formula group reached the optimal Omega‐3 Index level of > 8%, and the clinical relevance of the observed changes remains uncertain.

These findings may be explained by the lower baseline omega‐3 status of Indonesian toddlers and the relatively low DHA intake provided by the study formulas. While the European Food Safety Authority recommends 250 mg/day of DHA for this age group, the intervention dose was limited to 50 mg/day—the maximum achievable using available commercial formulas—even with two to three daily servings.

In comparison, a previous study in Malaysian toddlers using a 250 mg/day dose of microencapsulated DHA for 4 weeks achieved Omega‐3 Index values above 8% (Ghasemi Fard et al. 2020). Despite similar baseline saturated fat levels, Indonesian toddlers had lower baseline n‐3 PUFA (2.4%), DHA (1.6%), and Omega‐3 Index (2.4%) compared with Malaysian toddlers (4.7%, 3.3%, and 6.5%, respectively), and higher monounsaturated fat levels. These differences likely reflect population‐specific dietary patterns or metabolism and highlight the need for tailored nutritional strategies.

Several factors influence omega‐3 fatty acid bioavailability, including fat content, oil source, and gastrointestinal processing (Ghasemifard et al. 2014; Ghasemifard, Hermon, et al. 2015). While these variables are well documented in adults, limited data exist for young children. Moreover, frequent, lower‐dose administration—used in this study—may affect absorption differently than single, higher‐dose intake (Ghasemifard, Hermon, et al. 2015; Ghasemifard, Sinclair, et al. 2015). Given the complexity of absorption and metabolism, blood n‐3 LC‐PUFA concentrations alone may not fully reflect bioavailability. Therefore, this study also considered fatty acid excretion to provide a more complete assessment. While fecal DHA excretion declined slightly over time, the change was not significant.

To account for intake variability, formula consumption was included as a covariate in all analyses. Additionally, we assessed the relative bioavailability of DHA, DHA apparent digestibility, and iAUC of blood DHA. Relative DHA bioavailability was higher in both DHA groups compared with control, but not significantly different between the two DHA groups. However, apparent DHA digestibility and iAUC for blood DHA were significantly higher in group A compared with group C.

Adherence was highest in the microencapsulated group and lowest in the wet‐mixed group, possibly due to palatability differences. Although sensory characteristics were not directly assessed in this study, prior research has shown that microencapsulation can improve the sensory profile of fortified products by masking off‐flavors associated with fish oils and enhancing product acceptability in pediatric populations. In addition to potential sensory benefits, microencapsulation has been reported to improve oxidative stability and digestive efficiency by modifying lipid droplet size and surface composition, thereby enhancing the action of digestive enzymes such as pancreatic lipase (Holmberg et al. 2000). While these mechanisms were not examined directly in the present study, they may help explain the higher adherence and greater DHA bioavailability observed in the microencapsulated group.

Key strengths of this study include its randomized design, use of commercially available formulas, and assessment of DHA status via blood and fecal biomarkers. However, the small sample size, limited DHA dosage, and focus on a single demographic group limit generalizability.

## 5 Conclusions

This study demonstrates that microencapsulation may be a more effective strategy for enhancing DHA bioavailability in Indonesian toddlers compared with wet‐mixed formulations. After the 8‐week intervention, toddlers consuming the microencapsulated DHA formula exhibited significantly higher blood DHA and Omega‐3 Index levels compared with the control group. Apparent DHA digestibility and the incremental area under the curve (iAUC) for blood DHA were also significantly greater in the microencapsulated group than in the wet‐mixed group.

Future research is needed to determine whether higher DHA dosages or longer intervention durations using commercially available products can increase the Omega‐3 Index to optimal levels exceeding 8%. Additionally, studies involving a broader range of socioeconomic backgrounds in Indonesia are warranted to establish whether DHA insufficiency is widespread or primarily concentrated in lower‐income groups.

## Author Contributions

**Diana Sunardi:** conceptualization (equal), formal analysis (equal), investigation (equal), methodology (equal), resources (equal), writing – original draft (equal). **Samaneh G. Fard:** conceptualization, methodology, validation, resources, writing – original draft, visualization, supervision, funding acquisition. **Mia Puspita Ratih:** data curation (equal), investigation (equal), methodology (equal), project administration (equal), supervision (equal), writing – review and editing (equal). **Aprilia Herawati:** data curation (equal), methodology (equal), project administration (equal), resources (equal), writing – review and editing (equal). **Glenn Elliott:** conceptualization (equal), funding acquisition (equal), resources (equal), writing – review and editing (equal). **Andrew J. Sinclair:** conceptualization (equal), data curation (equal), formal analysis (equal), supervision (equal), visualization (equal), writing – review and editing (equal).

## Conflicts of Interest

The authors S.G.F. and G.E. are employees of Nu‐Mega Ingredients Pty Ltd., a manufacturer and supplier of high DHA fish oil and microencapsulated high‐DHA fish oil powder. Nu‐Mega Ingredients Pty Ltd. provided salary support for these authors and funded the study but had no role in the analysis of blood and fecal fatty acid samples. The formulas used were purchased commercially and, based on supplier knowledge, were fortified with Nu‐Mega's high‐DHA tuna oil or microencapsulated high‐DHA tuna oil powder. Brand names are not disclosed to protect commercial confidentiality.

## Supporting information


**Table S1:** Mean whole blood fatty acid levels (%) in toddlers fed different formulas.
**Table S2:** Mean selected blood omega‐6 and omega‐3 fatty acid levels (%) in toddlers fed various formulas over 8 weeks.
**Table S3:** Average biweekly fecal weight (g) and fecal fat excretion (g).
**Table S4:** Mean selected omega‐6 and omega‐3 fatty acids content of toddler feces (mg/g wet weight) in all groups over the 8 weeks.

## Data Availability

All data generated or analyzed during this study are included in this published article and its Tables S1–S4.
