# Supplementary material for: Tuna Oil‐Enriched Toddler Formula Enhances DHA Status in Indonesian Toddlers
Source: Food Sci Nutr. 2025 Sep 5;13(9):e70838. doi: 10.1002/fsn3.70838 (PMC12412412; doi:10.1002/fsn3.70838)
Supplement: Supplementary file 1 — Table S1: Mean whole blood fatty acid levels (%) in toddlers fed different formulas. Table S2: Mean selected blood omega‐6 and omega‐3 fatty acid levels (%) in toddlers fed various formulas over 8 weeks. Table S3: Average biweekly fecal weight (g) and fecal fat excretion (g). Table S4: Mean selected omega‐6 and omega‐3 fatty acids content of toddler feces (mg/g wet weight) in all groups over the 8 weeks. [file FSN3-13-e70838-s001.docx]

**Supplementary Table 1.** Mean whole blood fatty acid levels (%) in toddlers fed different formulas

|  | **Group A** | | | | | **Group B** | | | | | **Group C** | | | | | *P _(Group)_* | | *P*  *_(Time)_* | *P*  *_(Interaction)_* |
| --- | --- | --- | --- | --- | --- | --- | --- | --- | --- | --- | --- | --- | --- | --- | --- | --- | --- | --- | --- |
|  | **W0** | **W2** | **W4** | **W6** | **W8** | **W0** | **W2** | **W4** | **W6** | **W8** | **W0** | **W2** | **W4** | **W6** | **W8** |  | |  |  |
| Total Saturated | 39.76 ± 0.24 | 38.66 ± 0.51 | 38.59 ± 0.46 ^a^ | 38.68 ± 0.77 | 38.12 ± 0.71 ^a^ | 39.63 ± 0.28 | 39.89 ± 0.27 | 39.65 ± 0.23 ^b^ | 40.05 ± 0.36 | 40.05 ± 0.36 ^b^ | 39.62 ± 0.27 ^A^ | 39.95 ± 0.34 ^A^ | 39.64 ± 0.21 ^A ab^ | 39.41 ± 0.31 ^AB^ | 38.46 ± 0.84 ^B ab^ | <0.001 | | 0.005 | 0.004 |
| Total Monounsaturated | 25.97 ± 0.39 A | 25.90 ± 0.45 A | 25.42 ± 0.49 A | 26.05 ± 0.43 B | 25.11 ± 0.52 AB | 25.97 ± 0.43 | 25.64 ± 0.44 | 26.09 ± 0.34 | 26.55 ± 0.51 | 26.55 ± 0.51 | 25.57 ± 0.57 A | 24.81 ± 0.39 A | 25.53 ± 0.47 A | 26.74 ± 0.58 B | 25.67 ± 0.54 AB | 0.357 | | 0.010 | 0.226 |
| Total n-6 PUFA | 31.47 ± 0.44 | 31.89 ± 0.48 | 31.96 ± 0.50 | 31.75 ± 0.56 | 32.68 ± 0.62 | 32.02 ± 0.50 A | 32.23 ± 0.90 A | 31.50 ± 0.38 A | 31.22 ± 0.52 B | 31.22 ± 0.52 B | 31.90 ± 0.53 | 32.53 ± 0.49 | 32.34± 0.49 | 31.04 ± 0.65 | 32.87 ± 0.61 | 0.185 | | 0.037 | 0.277 |
| Total n-3 PUFA | 2.41 ± 0.16 A | 2.71 ± 0.20 AB a | 2.80 ± 0.18 AB a | 2.74 ± 0.15 AB a | 3.01± 0.15 B a | 2.10 ± 0.06 | 2.10 ± 0.05 b | 2.09 ± 0.06 b | 1.89 ± 0.08 b | 1.89 ± 0.08 b | 2.50 ± 0.17 A | 2.71 ± 0.15 B ab | 2.74 ± 0.14 B ab | 2.44 ± 0.14 A ab | 2.81 ± 0.15 B ab | <0.001 | | <0.001 | <0.001 |
| Total Trans Fat | 0.39 ± 0.03 A | 0.47 ± 0.05 A | 0.53 ± 0.05 AB a | 0.70 ± 0.05 B a | 0.74 ± 0.05 B a | 0.39 ± 0.02 | 0.38 ± 0.04 | 0.47 ± 0.04 b | 0.44 ± 0.04 b | 0.44 ± 0.04 b | 0.42 ± 0.05 A | 0.51 ± 0.10 AB | 0.62 ± 0.04 B ab | 0.38 ± 0.03 A b | 0.56 ± 0.05 AB ab | | 0.002 | <0.001 | <0.001 |

Value expressed as mean ± SE. Group A: dry-blended formula with microencapsulated high-DHA fish oil powder; Group B: unfortified control formula; Group C: wet-mixed formula with high-DHA fish oil. OmegaQuant measured dried blood spot samples and the units for each fatty acid reported as a percentage of the total fatty acids measured. In each row, values with lowercase different letters differ significantly for groups at the same time point (among groups) and values with uppercase different letters differ significantly for time within the same group (*P*<0.05). n-6 PUFA: 18:2n-6, 20:2n-6, 22:2n-6, 18:3n-6, 20:3n-6, 20:4n-6, 22:4n-6, 24:4n-6, 24:5n-6, 22:5n-6; n-3 PUFA: 18:3n3, 20:3n-3, 22:3n-3, 18:4n-3, 20:4n-3, 20:5n-3, 22:5n-3, 24:5n-3, 24:6n-3, 22:6n-3.

**Supplementary Table 2**. Mean selected blood omega-6 and omega-3 fatty acid levels (%) in toddlers fed various formulas over 8 weeks

| **n-6 UFA** | **Group A** | | | | | **Group B** | | | | | **Group C** | | | | | *P _(Group)_* | *P*  *_(Time)_* | *P*  *_(Interaction)_* |
| --- | --- | --- | --- | --- | --- | --- | --- | --- | --- | --- | --- | --- | --- | --- | --- | --- | --- | --- |
|  | **W0** | **W2** | **W4** | **W6** | **W8** | **W0** | **W2** | **W4** | **W6** | **W8** | **W0** | **W2** | **W4** | **W6** | **W8** |  |  |  |
| C18.2n-6 | 20.25 ± 0.32 | 21.84 ± 0.65 a | 21.74 ± 0.73 a | 21.98 ± 0.97 | 22.16 ± 0.91 | 19.96 ± 0.31 | 20.18 ± 0.35 b | 19.62 ± 0.36 b | 20.09 ± 0.41 | 20.11 ± 0.41 | 20.67 ± 0.34 | 19.41 ± 0.78 ab | 19.31 ± 0.92 ab | 19.87 ± 1.13 | 20.26 ± 1.07 | 0.008 | 0.101 | 0.925 |
| C18:3n-6 | 0.23 ± 0.02 | 0.20 ± 0.02 | 0.20 ± 0.02 | 0.18 ± 0.02 | 0.20 ± 0.02 | 0.24 ± 0.02 | 0.24 ± 0.02 | 0.19 ± 0.02 | 0.22 ± 0.02 | 0.22 ± 0.02 | 0.19 ± 0.01 | 0.17 ± 0.02 | 0.19 ± 0.02 | 0.17 ± 0.02 | 0.19 ± 0.02 | 0.162 | 0.293 | 0.052 |
| C20:2n-6 | 0.23 ± 0.01 | 0.24 ± 0.01 | 0.22 ± 0.01 | 0.24 ± 0.01 | 0.24 ± 0.01 | 0.22 ± 0.01 A | 0.23 ± 0.01 A | 0.26 ± 0.01 B | 0.22 ± 0.01 A | 0.22 ± 0.01 A | 0.22 ± 0.01 AB | 0.23 ± 0.01 AB | 0.31 ± 0.01 B | 0.20 ± 0.01 A | 0.23 ± 0.01 AB | 0.797 | <0.001 | <0.001 |
| C20:3n-6 | 1.60 ± 0.07 | 1.57 ± 0.07 | 1.51 ± 0.07 | 1.43 ± 0.07 | 1.55 ± 0.07 | 1.59 ± 0.05 | 1.68 ± 0.06 | 1.45 ± 0.06 | 1.52 ± 0.07 | 1.52 ± 0.07 | 1.54 ± 0.05 | 1.51 ± 0.05 | 1.34 ± 0.06 | 1.35 ± 0.07 | 1.60 ± 0.06 | 0.591 | 0.178 | 0.001 |
| ARA  (20:4n-6) | 7.25 ± 0.25 | 7.25 ± 0.25 | 7.35 ± 0.26 | 6.96 ± 0.23 | 7.43 ± 0.28 | 7.84 ± 0.21 | 7.66 ± 0.19 | 7.86 ± 0.22 | 7.16 ± 0.22 | 7.16 ± 0.22 | 7.37 ± 0.23 | 7.62 ± 0.22 | 7.73 ± 0.24 | 6.81 ± 0.28 | 7.67 ± 0.22 | 0.649 | 0.275 | 0.164 |
| 22:4n-6 | 1.21 ± 0.05 A | 1.21 ± 0.04 A | 1.20 ± 0.05 A | 1.13 ± 0.05 B | 1.20 ± 0.06 A | 1.38 ± 0.05 | 1.36 ± 0.04 | 1.39 ± 0.05 | 1.22 ± 0.04 | 1.22 ± 0.04 | 1.22 ± 0.05 A | 1.31 ± 0.05 A | 1.41 ± 0.06 A | 1.13 ± 0.06 B | 1.34 ± 0.05 A | 0.074 | <0.001 | <0.001 |
| 22:5n-6 | 0.70 ± 0.03 A | 0.72 ± 0.03 A | 0.73 ± 0.04 A | 0.61 ± 0.03 B a | 0.65 ± 0.03 A | 0.76 ± 0.03 | 0.81 ± 0.03 | 0.80 ± 0.03 | 0.76 ± 0.03 b | 0.76 ± 0.03 | 0.71 ± 0.04 A | 0.80 ± 0.04 A | 0.71 ± 0.04 A | 0.59 ± 0.04 B a | 0.70 ± 0.03 A | 0.021 | <0.001 | <0.001 |
| **n-3 PUFA** | **Group A** | | | | | **Group B** | | | | | **Group C** | | | | | *P _(Group)_* | *P*  *_(Time)_* | *P*  *_(Interaction)_* |
|  | **W0** | **W2** | **W4** | **W6** | **W8** | **W0** | **W2** | **W4** | **W6** | **W8** | **W0** | **W2** | **W4** | **W6** | **W8** |  |  |  |
| ALA  (18:3n-3) | 0.21 ± 0.02 A a | 0.27 ± 0.02 B | 0.27 ± 0.03 B a | 0.26 ± 0.03 B | 0.27 ± 0.03 B | 0.16 ± 0.01 A b | 0.20 ± 0.01 B | 0.16 ± 0.01 A b | 0.19 ± 0.01 B | 0.19 ± 0.01 B | 0.22 ± 0.02 A a | 0.27 ± 0.02 B | 0.24 ± 0.02 AB ab | 0.30 ± 0.02 B | 0.21 ± 0.02 A | <0.001 | 0.008 | <0.001 |
| EPA  (20:5n-3) | 0.15 ± 0.02 | 0.18 ± 0.02 | 0.16 ± 0.02 a | 0.13 ± 0.01 | 0.15 ± 0.02 | 0.12 ± 0.01 A | 0.13 ± 0.01 A | 0.13 ± 0.01 A b | 0.09 ± 0.01 B | 0.09 ± 0.01 B | 0.13 ± 0.02 | 0.14 ± 0.01 | 0.18 ± 0.01 a | 0.14 ± 0.01 | 0.12 ± 0.01 | 0.007 | <0.001 | 0.004 |
| DPA  (22:5n-3) | 0.45 ± 0.02 | 0.48 ± 0.03 | 0.51 ± 0.03 | 0.43 ± 0.02 | 0.48 ± 0.02 | 0.42 ± 0.01 A | 0.44 ± 0.02 B | 0.44 ± 0.02 B | 0.39 ± 0.02 A | 0.39 ± 0.02 A | 0.45 ± 0.03 | 0.46 ± 0.02 | 0.56 ± 0.02 | 0.40 ± 0.02 | 0.45 ± 0.02 | 0.042 | <0.001 | <0.001 |
| DHA  (22:6n-3) | 1.60 ± 0.12 A | 1.76 ± 0.15 AB ab | 1.83 ± 0.13 B | 1.91 ± 0.11 B a | 2.09 ± 0.13 B a | 1.37 ± 0.05 | 1.33 ± 0.04 b | 1.36 ± 0.05 | 1.22 ± 0.06 b | 1.22 ± 0.06 b | 1.70 ± 0.12 A | 1.86 ± 0.13 B a | 1.79 ± 0.13 A | 1.61 ± 0.12 A ab | 2.05 ± 0.13 B ab | 0.000 | 0.000 | 0.000 |
| Omega-3 Index | 3.33 ± 0.12 A | 3.56 ± 0.14 AB a | 3.59 ± 0.14 AB | 3.65 ± 0.15 AB a | 3.90 ± 0.15 B a | 3.02 ± 0.11 | 3.00 ± 0.12 b | 3.01 ± 0.12 | 2.81 ± 0.12 b | 2.81 ± 0.13 b | 3.36 ± 0.13 A | 3.44 ± 0.28 B ab | 3.54 ± 0.32 B | 3.26 ± 0.33 A ab | 3.66 ± 0.34 B ab | <0.001 | <0.001 | <0.001 |
| n-6:n-3 | 14.58± 0.68 A | 13.16 ± 0.72 A | 12.73 ± 0.66 AB | 12.69 ± 0.65 AB a | 11.82 ± 0.60 B a | 15.72 ± 0.65 | 15.41 ± 0.33 | 15.38 ± 0.54 | 16.98 ± 0.49 b | 16.98 ± 0.49 b | 14.19 ± 0.7 A | 12.83 ± 0.61 AB | 12.43 ± 0.74 B | 13.64 ± 0.63 AB ab | 12.51 ± 0.66 B ab | <0.001 | <0.001 | <0.001 |
| AA:EPA | 72.54 ± 8.20 | 53.79 ± 5.89 | 67.28 ± 8.88 | 76.17 ± 10.26 | 70.60 ± 8.00 | 71.52 ± 4.21 A | 69.76 ± 5.46 A | 72.53 ± 7.32 A | 96.27 ± 9.63 B | 96.27 ± 9.63 B | 78.32 ± 8.26 | 65.46 ± 5.48 | 47.34 ± 3.89 | 73.36 ± 8.77 | 71.75 ± 4.91 | 0.052 | <0.001 | <0.001 |

Value expressed as mean ± SE. Group A: dry-blended formula with microencapsulated high-DHA fish oil powder; Group B: unfortified control formula; Group C: wet-mixed formula with high-DHA fish oil. OmegaQuant measured dried blood spot samples and the units for each fatty acid reported as a percentage of the total fatty acids measured. In each row, values with lowercase different letters differ significantly for groups at the same time point (among groups) and values with uppercase different letters differ significantly for time within the same group (*P*<0.05).

**Supplementary Table 3.** Average biweekly fecal weight (g) and fecal fat excretion (g)

| **Time/ Group** | **Group A** | **Group B** | **Group C** | P  _(Group)_ | P  _(Time)_ | P  _(Interaction)_ |
| --- | --- | --- | --- | --- | --- | --- |
| Average Biweekly Fecal Weight (g) | | | | |  |  |
| W0 | 76.94 ± 5.02 | 72.35 ± 4.80 | 76.63 ± 5.31 | 0.416 | 0.551 | 0.886 |
| W2 | 67.58 ± 3.48 | 71.96 ± 1.86 | 74.66 ± 4.13 |  |  |  |
| W4 | 70.07 ± 4.13 | 69.41 ± 2.02 | 69.60 ± 5.17 |  |  |  |
| W6 | 70.08 ± 4.15 | 72.02 ± 1.74 | 71.57 ± 4.81 |  |  |  |
| W8 | 67.02 ± 4.22 | 72.05 ± 1.87 | 74.98 ± 4.89 |  |  |  |
|  |  |  |  |  |  |  |
| Average Biweekly Fecal Fat Excretion (g) | | | | |  |  |
| W0 | 3.24 ± 0.34 | 2.25 ± 0.25 | 1.89 ± 0.27 | 0.728 | 0.226 | 0.094 |
| W2 | 2.21 ± 0.23 | 2.42 ± 0.28 | 2.17 ± 0.18 |  |  |  |
| W4 | 2.33 ± 0.22 | 1.90 ± 0.20 | 1.43 ± 0.20 |  |  |  |
| W6 | 2.36 ± 0.25 | 1.98 ± 0.20 | 1.71 ± 0.25 |  |  |  |
| W8 | 2.97 ± 0.41 | 2.01 ± 0.19 | 1.45 ± 0.13 |  |  |  |
|  |  |  |  |  |  |  |

Value expressed as mean ± SE. Group A: dry-blended formula with microencapsulated high-DHA fish oil powder; Group B: unfortified control formula; Group C: wet-mixed formula with high-DHA fish oil.

**Supplementary Table 4**. Mean selected omega-6 and omega-3 fatty acids content of toddler faeces (mg/g wet weight) in all groups over the 8 weeks

|  | **Group A** | | | | | **Group B** | | | | | **Group C** | | | | | *P_(Group)_* | *P _(Time)_* | *P _Interaction)_* |
| --- | --- | --- | --- | --- | --- | --- | --- | --- | --- | --- | --- | --- | --- | --- | --- | --- | --- | --- |
| **n-6 UFA** (mg/g) | **W0** ^2^ | **W2** | **W4** | **W6** | **W8** | **W0** | **W2** | **W4** | **W6** | **W8** | **W0** | **W2** | **W4** | **W6** | **W8** |  |  |  |
| C18:2n-6 | 1.95 ± 0.61 | 3.29 ± 1.21 | 1.98 ± 0.98 | 2.49 ± 0.58 | 5.34 ± 1.51 a | 1.61 ± 0.48 | 4.47 ± 1.52 | 2.09 ± 0.66 | 0.81 ± 0.22 | 0.80 ± 0.21 b | 1.67 ± 0.88 | 1.48 ± 0.42 | 1.40 ± 0.54 | 1.80 ± 0.73 | 1.18 ± 0.37 ab | 0.019 | 0.156 | 0.016 |
| C18:3n-6 | 0.012 ± 0.001 | 0.011 ± 0.004 | 0.013 ± 0.003 | 0.021 ± 0.005 | 0.022 ± 0.004 a | 0.010 ± 0.001 | 0.009 ± 0.002 | 0.009 ± 0.001 | 0.009± 0.002 | 0.009 ± 0.002 b | 0.006 ± 0.001 | 0.009 ± 0.004 | 0.004 ± 0.004 | 0.006 ± 0.006 | 0.005 ± 0.003 b | 0.004 | 0.203 | 0.338 |
| C20:2n-6 | 0.007 ± 0.001 | 0.006 ± 0.002 | 0.007 ± 0.002 | 0.007 ± 0.002 | 0.010 ± 0.002 | 0.006 ± 0.001 | 0.006 ± 0.002 | 0.005 ± 0.001 | 0.007 ± 0.000 | 0.007 ± 0.001 | 0.008 ± 0.001 | 0.010 ± 0.002 | 0.009 ± 0.002 | 0.006 ± 0.002 | 0.006 ± 0.001 | 0.052 | 0.851 | 0.173 |
| C20:3n-6 | 0.017 ± 0.004 | 0.005 ± 0.005 | 0.007 ± 0.001 | 0.001 ± 0.006 | 0.011 ± 0.006 | 0.019 ± 0.003 | 0.016 ± 0.003 | 0.017 ± 0.004 | 0.019 ± 0.002 | 0.018 ± 0.003 | 0.024 ± 0.004 | 0.028 ± 0.006 | 0.046 ± 0.012 | 0.044 ± 0.007 | 0.027 ± 0.008 | 0.688 | 0.178 | 0.155 |
| ARA (20:4n-6) | 0.016 ± 0.003 | 0.014 ± 0.002 | 0.018 ± 0.003 | 0.018 ± 0.005 | 0.019 ± 0.004 | 0.020 ± 0.002 | 0.016 ± 0.001 | 0.014 ± 0.001 | 0.018 ± 0.002 | 0.018 ± 0.002 | 0.019 ± 0.003 | 0.015 ± 0.003 | 0.016 ± 0.004 | 0.021 ± 0.006 | 0.017 ± 0.005 | 0.672 | 0.012 3 | 0.510 |
| 22:4n-6 | 0.005 ± 0.001 | 0.002 ± 0.001 | 0.004 ± 0.003 | 0.003 ± 0.008 | 0.006 ± 0.002 | 0.006 ± 0.001 A | 0.004 ± 0.000 B | 0.005 ± 0.001 B | 0.005 ± 0.003 B | 0.005 ± 0.003 B | 0.008 ± 0.001 | 0.008 ± 0.001 | 0.010 ± 0.003 | 0.009 ± 0.009 | 0.008 ± 0.002 | 0.034 | 0.136 | 0.373 |
| 22:5n-6 | 0.005 ± 0.001 | 0.002 ± 0.001 | 0.003 ± 0.001 | 0.004 ± 0.001 | 0.006 ± 0.001 | 0.005 ± 0.001 | 0.004 ± 0.000 | 0.003 ± 0.000 | 0.004 ± 0.001 | 0.004 ± 0.000 | 0.004 ± 0.001 | 0.007 ± 0.001 | 0.005 ± 0.001 | 0.004 ± 0.001 | 0.003 ± 0.001 | 0.319 | 0.323 | 0.396 |
|  | **Group A** | | | | | **Group B** | | | | | **Group C** | | | | | *P_(Group)_* | *P _(Time)_* | *P _Interaction)_* |
| **n-3 PUFA** (mg/g) | **W0** | **W2** | **W4** | **W6** | **W8** | **W0** | **W2** | **W4** | **W6** | **W8** | **W0** | **W2** | **W4** | **W6** | **W8** |  |  |  |
| ALA (mg/g) | 0.113 ± 0.075 | 0.229 ± 0.130 | 0.265 ± 0.199 | 0.136 ± 0.124 | 0.085 ± 0.059 | 0.067 ± 0.068 | 0.205 ± 0.069 | 0.202 ± 0.097 | 0.031 ± 0.052 | 0.030 ± 0.026 | 0.147 ± 0.075 | 0.033 ± 0.154 | 0.055 ± 0.249 | 0.067 ± 0.142 | 0.068 ± 0.069 | 0.912 | 0.059 | 0.874 |
| EPA (mg/g) | 0.002 ± 0.000 | 0.003 ± 0.001 | 0.002 ± 0.001 | 0.002 ± 0.003 | 0.003 ± 0.000 | 0.002 ± 0.000 A | 0.002 ± 0.000 A | 0.001 ± 0.000 B | 0.001 ± 0.001 B | 0.001 ± 0.000 B | 0.003 ± 0.000 | 0.003 ± 0.001 | 0.002 ± 0.001 | 0.001 ± 0.001 | 0.001 ± 0.001 | 0.027 | 0.296 | 0.109 |
| EPA excretion (mg/day) | 0.18 ± 0.05 | 0.22 ± 0.10 | 0.17 ± 0.09 | 0.15 ± 0.25 | 0.20 ± 0.03 | 0.15 ± 0.05 A | 0.14 ± 0.05 A | 0.09 ± 0.04 B | 0.10 ± 0.09 B | 0.11 ± 0.01 B | 0.25 ± 0.05 A | 0.21 ± 0.12 A | 0.16 ± 0.12 AB | 0.16 ± 0.27 AB | 0.08 ± 0.03 B | 0.022 | 0.279 | 0.145 |
| DPA (mg/g) | 0.006 ± 0.002 | 0.005 ± 0.003 | 0.004 ± 0.006 | 0.004 ± 0.003 | 0.007 ± 0.005 | 0.005 ± 0.002 | 0.004 ± 0.001 | 0.004 ± 0.003 | 0.004 ± 0.001 | 0.004 ± 0.002 | 0.009 ± 0.002 | 0.008 ± 0.004 | 0.007 ± 0.006 | 0.008 ± 0.001 | 0.007 ± 0.000 | 0.318 | 0.667 | 0.133 |
| DPA excretion (mg/day) | 0.58 ± 0.14 | 0.34 ± 0.27 | 0.15 ± 0.14 | 0.32 ± 0.23 | 0.47 ± 0.32 | 0.40 ± 0.14 | 0.29 ± 0.14 | 0.26 ± 0.22 | 0.35 ± 0.09 | 0.36 ± 0.14 | 0.62 ± 0.15 | 0.67 ± 0.32 | 0.62 ± 0.17 | 0.71 ± 0.27 | 0.50 ± 0.38 | 0.338 | 0.747 | 0.164 |
| DHA (mg/g) | 0.008 ± 0.001 | 0.006 ± 0.002 | 0.007 ± 0.002 | 0.009 ± 0.001 | 0.009 ± 0.001 | 0.008 ± 0.001 | 0.006 ± 0.001 | 0.006 ± 0.000 | 0.006 ± 0.001 | 0.007 ± 0.000 | 0.009 ± 0.001 | 0.009 ± 0.001 | 0.008 ± 0.002 | 0.008 ± 0.003 | 0.008 ± 0.003 | 0.122 | 0.139 | 0.259 |
| DHA excretion (mg/day) | 0.81 ± 0.17 | 0.41 ± 0.18 | 0.56 ± 0.14 | 0.72 ± 0.18 | 0.73 ± 0.16 | 0.64 ± 0.16 | 0.47 ± 0.09 | 0.45 ± 0.07 | 0.54 ± 0.21 | 0.57 ± 0.07 | 0.78 ± 0.18 | 0.74 ± 0.21 | 0.56 ± 0.17 | 0.56 ± 0.21 | 0.49 ± 0.19 | 0.245 | 0.255 | 0.442 |

Value expressed as mean ± SE. Group A: dry-blended formula with microencapsulated high-DHA fish oil powder; Group B: unfortified control formula; Group C: wet-mixed formula with high-DHA fish oil. In each row, values with lowercase different letters differ significantly for groups at the same time point (among groups) and values with uppercase different letters differ significantly for time within the same group (P<0.05). 1 The fecal collection was conducted over one week, given that the wet fecal weight remained consistent across different time points in each group, we assumed that the weekly wet weight of feces serves as an average representation for the biweekly period. 2 Feces for the baseline (W0) were collected on the day before the study commenced (day -1). 3 A two-way ANOVA revealed a significant difference between groups in curtain time point; however, this difference was not detected in the post-hoc Tukey HSD test.
